# East China Sea increasingly gains limiting nutrient P from South China Sea

**DOI:** 10.1038/s41598-019-42020-4

**Published:** 2019-04-04

**Authors:** Ting-Hsuan Huang, Chen-Tung Arthur Chen, Jay Lee, Chau-Ron Wu, You-Lin Wang, Yan Bai, Xianqiang He, Shu-Lun Wang, Selvaraj Kandasamy, Jiann-Yuh Lou, Ben-Jei Tsuang, Hsien-Wen Chen, Ruo-Shan Tseng, Yiing Jang Yang

**Affiliations:** 1 Department of Oceanography, National Sun Yat-sen University, Kaohsiung, Taiwan; 2 Taiwan Ocean Research Institute, National Applied Research Laboratories, Kaohsiung, Taiwan; 3 Department of Earth Sciences, National Taiwan Normal University, Taipei, Taiwan; 4 State Key Laboratory of Satellite Ocean Environment Dynamics, Second Institute of Oceanography, Ministry of Natural Resources, Hangzhou, China; 5 Ocean College, Zhejiang University, Zhoushan, China; 6 Department of Marine Environmental Engineering, National Kaohsiung University of Science and Technology, Kaohsiung, Taiwan; 7 Department of Geological Oceanography and State Key Laboratory of Marine Environmental Science, Xiamen University, Xiamen, China; 8 Department of Marine Science, R.O.C. Naval Academy, Kaohsiung, Taiwan; 9 Department of Environmental Engineering, Innovation and Development Center of Sustainable Agriculture (IDCSA), National Chung Hsing University, Taichung, Taiwan; 10 Department of Maritime Police, Central Police University, Taoyuan, Taiwan; 11 Institute of Oceanography, National Taiwan University, Taipei, Taiwan

## Abstract

The Taiwan Strait (TS) directly connects two of the richest fishing grounds in the world - the East China Sea (ECS) and the South China Sea (SCS). Carbon and nutrient supplies are essential for primary production and the Yangtze River is an important source for the ECS. However the ECS is severely P-limited. The TS transports an order of magnitude more carbon and a factor of two more phosphate (P) to the ECS than the Yangtze River does. To evaluate the temporal variability of these supplies, the total alkalinity (TA), dissolved inorganic carbon (DIC), nitrate plus nitrite (N), P, and silicate (Si) fluxes through the TS were estimated using empirical equations for these parameters and the current velocity, which was estimated using the Hybrid Coordinate Ocean Model (HYCOM). These empirical equations were derived from *in situ* salinity and temperature and measured chemical concentrations that were collected during 57 cruises (1995–2014) with a total of 2096 bottle samples. The 24-month moving averages of water, carbon, and nutrient fluxes significantly increase with time, so does the satellite chlorophyll *a* concentration. More importantly, the increased supply of the badly needed P from the TS is more than that from the Yangtze River.

## Introduction

The East China Sea (ECS) and the South China Sea (SCS) are two of the richest fishing grounds in the world and are directly connected by the Taiwan Strait (TS). The large fishery yield is supported by primary production, which is related to nutrient supplies from various sources in this area, including currents, upwelling, eddies, rivers, submarine groundwater discharge, and anthropogenic input^1–11^. Approximately 0.3 billion people live along the southeast coast of China and consume 15% of China’s fresh water resources^12,13^. As a result of river runoff, the increasing domestic waste and fertilizer use have increased the area of eutrophication, increasing frequencies of harmful algal and jellyfish blooms in the coastal area^14–16^.

The two largest rivers in China are the Yangtze River (YR; Changjiang) and the Pearl River (Zhujiang), which flow into the ECS and the SCS, respectively. The ratios of nitrogen to phosphorus (N/P) in the YR and the Pearl River are as high as 250 and 340, respectively^17,18^. These ratios are much higher than the marine biological consummation ratio of 16 (Redfield ratio). Primary production is commonly limited by N concentration in the world oceans, but high riverine N/P ratios make large plume-affected area in the ECS P-limited^19–22^. The altered nutrient ratios in coastal area are related to jellyfish blooms and harmful algal blooms, and associated red tides were the most frequent major marine disasters in China from 2004 to 2015^23^. Apart from the riverine nutrient inputs into the ECS and the SCS, interactions between these two marginal seas cause them to exchange nutrients.

**Figure 1:**
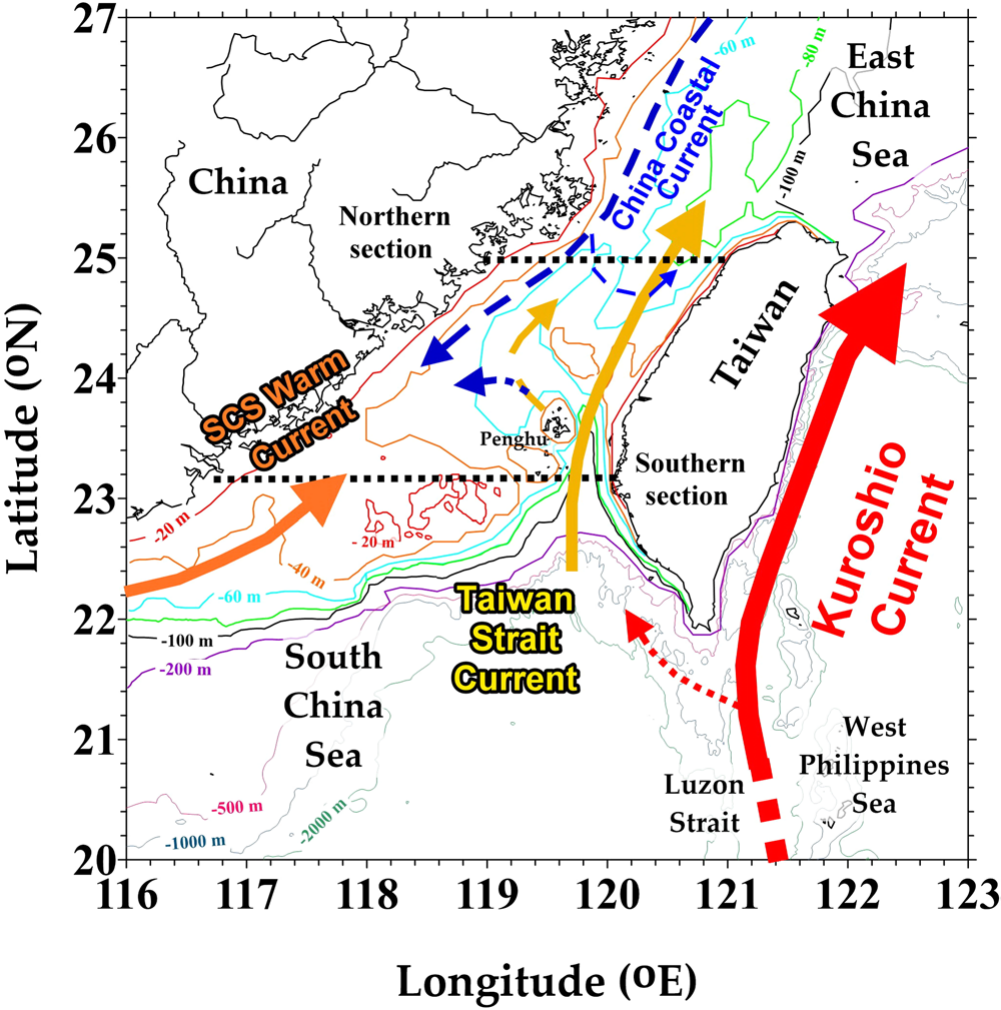
Study area and schematic current. Blue dashed lines represent currents that only exist during northeast monsoon periods. Black dashed lines delineate northern and southern sections in the TS.

The TS directly transports material between the ECS and the SCS, and importantly affects the carbon and nutrient budgets in the ECS and northern SCS. The main water masses in the TS are SCS water, West Philippines Sea water (WPS, Kuroshio Branch), and the southward China Coastal Current (CCC, existing only during the northeast monsoon period in the western TS). West of Taiwan is the northward outgoing flow from the SCS, which is the SCS Warm Current, which mixes with WPS water in the TS, and becomes the Taiwan Strait Current (TSC; Fig. 1)^24^. The current system in the TS is affected not only by seasonal variations but also by large-scale climatic events and patterns like El Niño, La Niña, and the Pacific Decadal Oscillation (PDO)^25–28^.

The interactions among the ECS, SCS, and TS also vary with monsoons and climate events^29^, further affecting the transport of carbon and nutrients. However, published inorganic carbon and nutrient transports in the TS were estimated by considering only a few cruise data. To understand the exchanges between the ECS and the SCS, the study area covers cross-sections at both ends of the TS. Compared with our preliminary study which investigated the southeastern corner of the TS^30^, this investigation focuses on the interaction between the ECS and the SCS, and mechanisms affecting long-term variations in the TS.

## Results and Discussion

### Water and chemical transports

**Figure 2:**
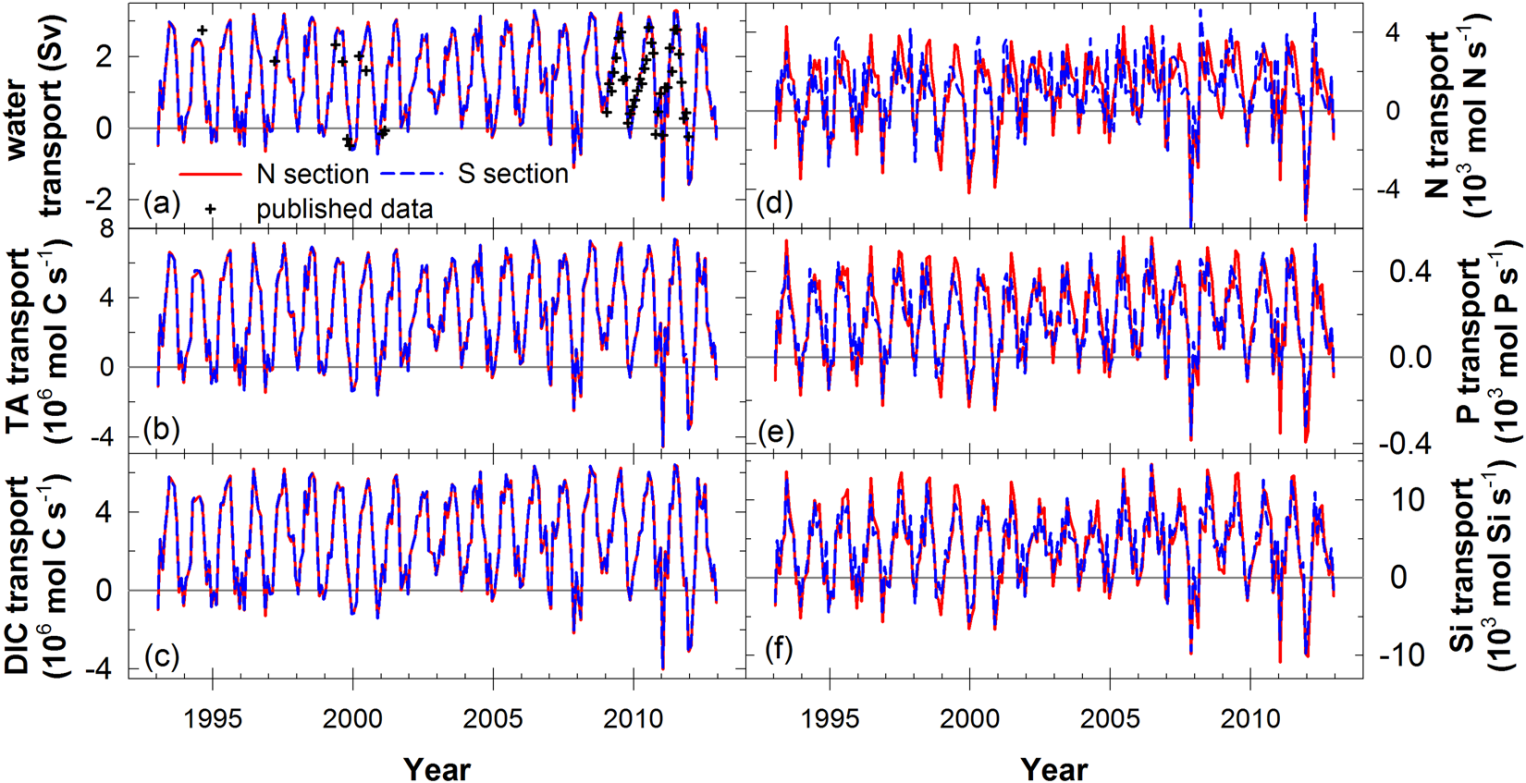
Time series of (**a**) water, (**b**) TA, (**c**) DIC, (**d**) N, (**e**) P, and (**f**) Si model-calculated transports. Positive and negative values represent the northward and southward transports, respectively. Red and blue colors represent fluxes in the northern section and the southern section, respectively. The cross symbols in (**a**) represent the data from published articles^31–33^.

Figure 2 presents the water, total alkalinity (TA), dissolved inorganic carbon (DIC), N, P, and silicate (Si) transports in the Northern section (N section) and the Southern section (S section) of the TS from 1993 to 2012. These transports exhibit clear seasonal and interannual variations. Compared with the published data, the water transports from HYCOM are similar to the estimation based on measurements (Fig. 2a, Supplementary Fig. S4c) from 1993 to 2010^31–33^. The transports in the N section and S section are almost equal, except for some slight differences in the nutrient transports, suggesting that the main controlling process in the TS is physical transfer. Similar values in the N section and S section also imply that the biological uptake and decomposition are small compared to the horizontal transports in the TS. Overall, the water, TA, DIC, N, P, and Si transports in the TS are 1.42 ± 0.16 Sv, 3.21 ± 0.37 mmol C s^−1^, 2.78 ± 0.32 mmol C s^−1^, 1.04 ± 0.43 kmol N s^−1^, 0.19 ± 0.033 kmol P s^−1^, and 4.22 ± 0.86 kmol Si s^−1^, respectively (Table 1). The estimated water transport in this investigation is in the reported range of estimated water transport in the TS^33–36^ (1.2–2.0 Sv). In published investigations, the TA, N, P, and Si transports through the TS to the ECS were 0.794 mmol C s^−1^, 0.71–6.4 kmol N s^−1^, 0.07–0.45 kmol P s^−1^, and 1.8–16.3 kmol Si s^−1^, respectively^1,37–40^ (Table 1). Those estimates were calculated using chemical data from one or only a few cruises, and so may exhibit higher uncertainty than the data in this study based on 57 cruises. Our pervious study reported that the water, TA, DIC, N, P, and Si transports in the deepest part of the TS, Penghu Channel, are 0.81 ± 0.34 Sv, 1.87 ± 0.79 mmol C s^−1^, 1.65 ± 0.71 mmol C s^−1^, 1.20 ± 0.99 kmol N s^−1^, 0.13 ± 0.085 kmol P s^−1^, and 3.06 ± 1.83 kmol Si s^−1 ^^30^. Generally, the water and chemical transports in the TS are higher than those in the Penghu Channel except the N transport, and that reflects the substantial southward N transport by the CCC in the TS.

**Table 1:** TA, DIC, N, P, and Si fluxes (kmol s^−1^) and N/P ratios in various waters.

water source	TA	DIC	N	P	Si	N/P	References
Yangtze River (Changjiang)	69.8		3.2	0.029	5.1	111.1	Chen and Wang^1^
				0.017			Fang^39^
			1.4	0.0054	2.7	262.2	Li *et al*.^17^
			1.8	0.0095	2.7	185.5	Zhang *et al*.^40^
		34.2					Zhang *et al*.^64^
Taiwan Strait	794		0.7	0.07	1.8	10.0	Chen and Wang^1^
			1.9	0.25	8.4	7.6	Liu *et al*.^65^
			1.4	0.25		5.6	Chung *et al*.^38^
				0.45			Fang^39^
			6.4	0.41	16.3	15.7	Zhang *et al*.^40^
	3120	2777	1.0	0.19	4.22	5.3	this study
Kuroshio intrusion into the ECS	2754		4.7	0.3	9.7	15.7	Chen and Wang^1^
				0.8			Fang^39^
			9.0	0.65	15.0	13.8	Zhang *et al*.^40^
			9.4	0.7	18.2	13.4	Zhao and Guo^66^
		3710					Qu *et al*.^67^
Kuroshio	55943	51133	173.5	11.75	396.3	14.8	Chen *et al*.^68^
			170.8	12.52		13.6	Guo *et al*.^69^
			204.8				Guo *et al*.^70^
			181.8	17.3		10.5	Chen *et al*.^71^

The main nutrient sources in the ECS are Kuroshio, the Taiwan Warm Current (the extension of the TSC), and the YR. The N and Si transports in the TS are of the same order as those from the YR. However, the P transport in the TS is 10 to 100 times that from the YR (Table 1). The N/P ratio of the YR is around 100 to 200 or even higher, and is much higher than the bio-utilization ratio of 16. The excess nitrate from rivers makes the ECS a P-limited environment. Therefore, the supply of P to the ECS is the main factor that controls phytoplankton productivity therein. The N/P ratio in the TSC is notably low (Table 1). This fact is consistent with the low ratio in the surface water in the North Pacific Ocean^41^, the SCS basin^42^, and the Luzon Strait^43^. Regardless of whether the low N/P ratio in the TS arises from the source waters or faster regeneration of P than of N^40,44^, the excess of P over N compensates for the P deficiency in the ECS. The input transport from the Kuroshio, although two to four times the transport in the TS, is associated with an N/P ratio that is close to the Redfield ratio (Table 1). As a result, the Kuroshio input does not resolve the P-limiting problem in the ECS.

### Annual variation

**Figure 3:**
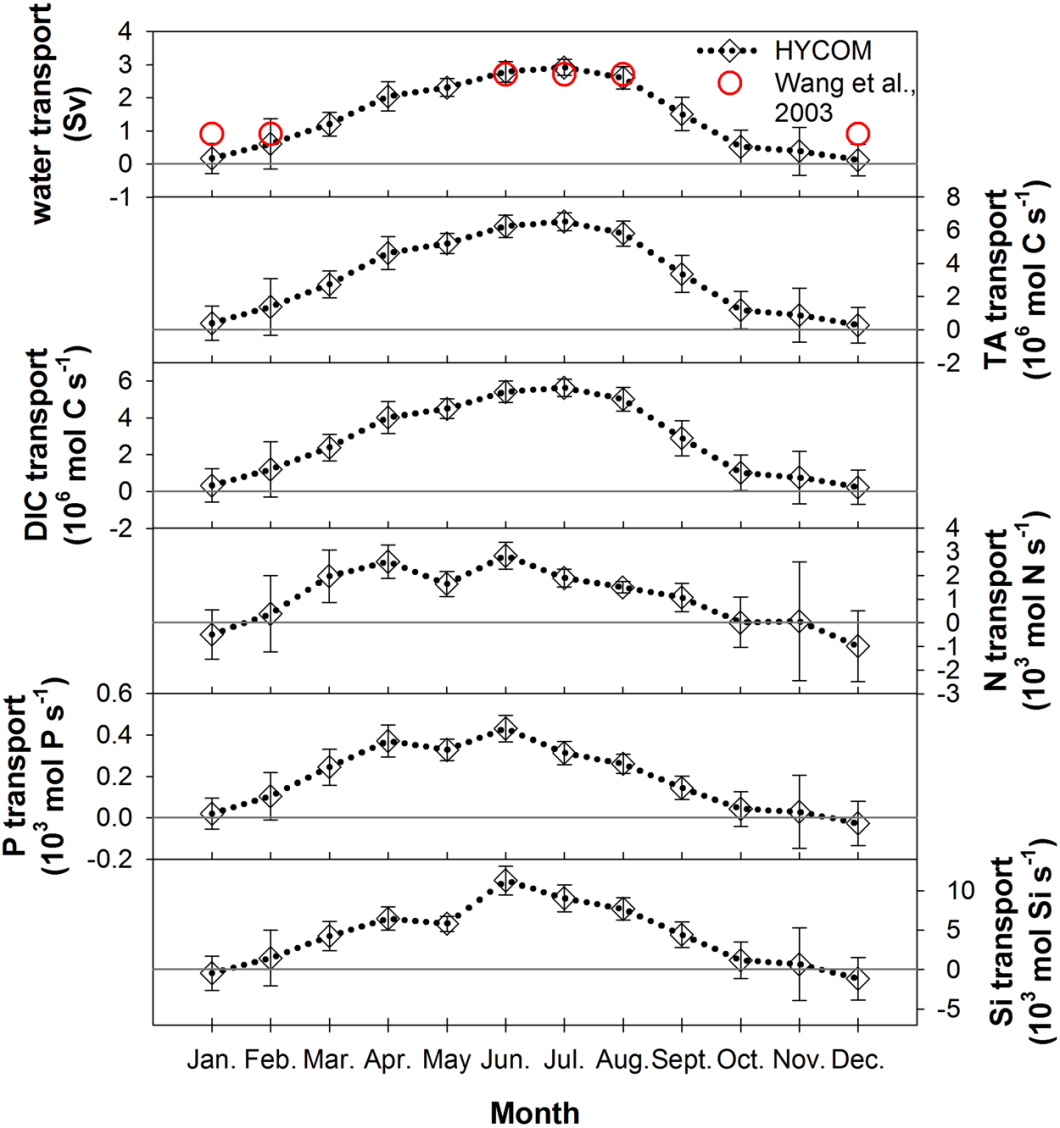
Monthly average (**a**) water, (**b**) TA, (**c**) DIC, (**d**) N, (**e**) P, and (**f**) Si model-calculated transports. Positive and negative values represent northward and southward transports, respectively. The error bars of parameters represent the monthly variation ranges from 1993 to 2010. The circle symbols in (**a**) are the data from Wang *et al*.^36^.

The seasonal variations in the monthly transports of water, TA, DIC, N, P, and Si (Fig. 3) are associated with the monsoons and the variations in the water mass mixing ratios in the TS. Generally, water flux increases from January and reaches its highest value in July, decreasing to December. The lowest water transports are close to zero during December and January, and the highest value in July is almost 3.0 Sv. Compared with the published shipboard ADCP (Acoustic Doppler current profile)-averaged results between 1999 to 2001, the seasonal values are similar with the HYCOM results (Fig. 3a)^36^. The patterns of TA and DIC transports are consistent with the water transport in the TS. However, the patterns of nutrient transports slightly differ from the water transport. They are also lowest in December and January, but most of them are negative except the P transport in January, indicating that the net nutrient transports are southward. Although the water transport is close to zero, the southward nutrient transports from the CCC exceed the northward nutrient transports in December and January. Han *et al*.^45^ estimated the southward N transport to be 11.12 ± 7.96 × 10^9^ mol season^−1^ (1.4 kmol N s^−1^) through the TS to the northern SCS from December to February. The averaged net N, P, and Si transports in this investigation are −0.98 kmol N s^−1^, −0.027 kmol P s^−1^, and −1.17 kmol Si s^−1^ in December. Interestingly, the N/P ratio of 37 exceeds the Redfield ratio. Based on the assumption that primary production is consistent with the Redfield ratio; an excess N of approximately 0.55 kmol N s^−1^ would be left after all of the P has been consumed, and N could fix 3.65 kmol C s^−1^. As surmised by Han *et al*.,^41^ primary production in the winter in the northern SCS is in the range 0.51–0.82 gC m^−2^ d^−1 ^^46,47^, and the area of the northern SCS that is shallower than 100 m is approximately 8.8 × 10^4^ km^2^. The excess N from the ECS can support 5–8% of the primary production in the northern SCS during December.

In the TS, the nutrient transports are highest during the late spring to early summer (Fig. 3d–f) whereas the water, TA and DIC transports are highest in July. This situation may be associated with the mixed layer depth and the distributions of chemical parameters in the SCS. In winter, the disturbance of the SCS surface seawater by the strong northeast monsoon makes the mixed layer deeper than in summer. Meanwhile, the surface N and P concentrations of the SCS in winter can be two to 15 times of those in summer^42,48^. In contrast, the seasonal differences in the average TA and DIC concentrations of the SCS in the mixed layer are only 1.5%^49^. The high surface nutrient concentrations of the SCS decrease from winter to summer while stratification and northward transport to the TS strengthen from winter to summer. In the TS, even though the water transport is highest in July, the nutrient transports in July are lower than those in late spring and early summer because the nutrient concentrations are lower.

### Interannual trend

**Figure 4:**
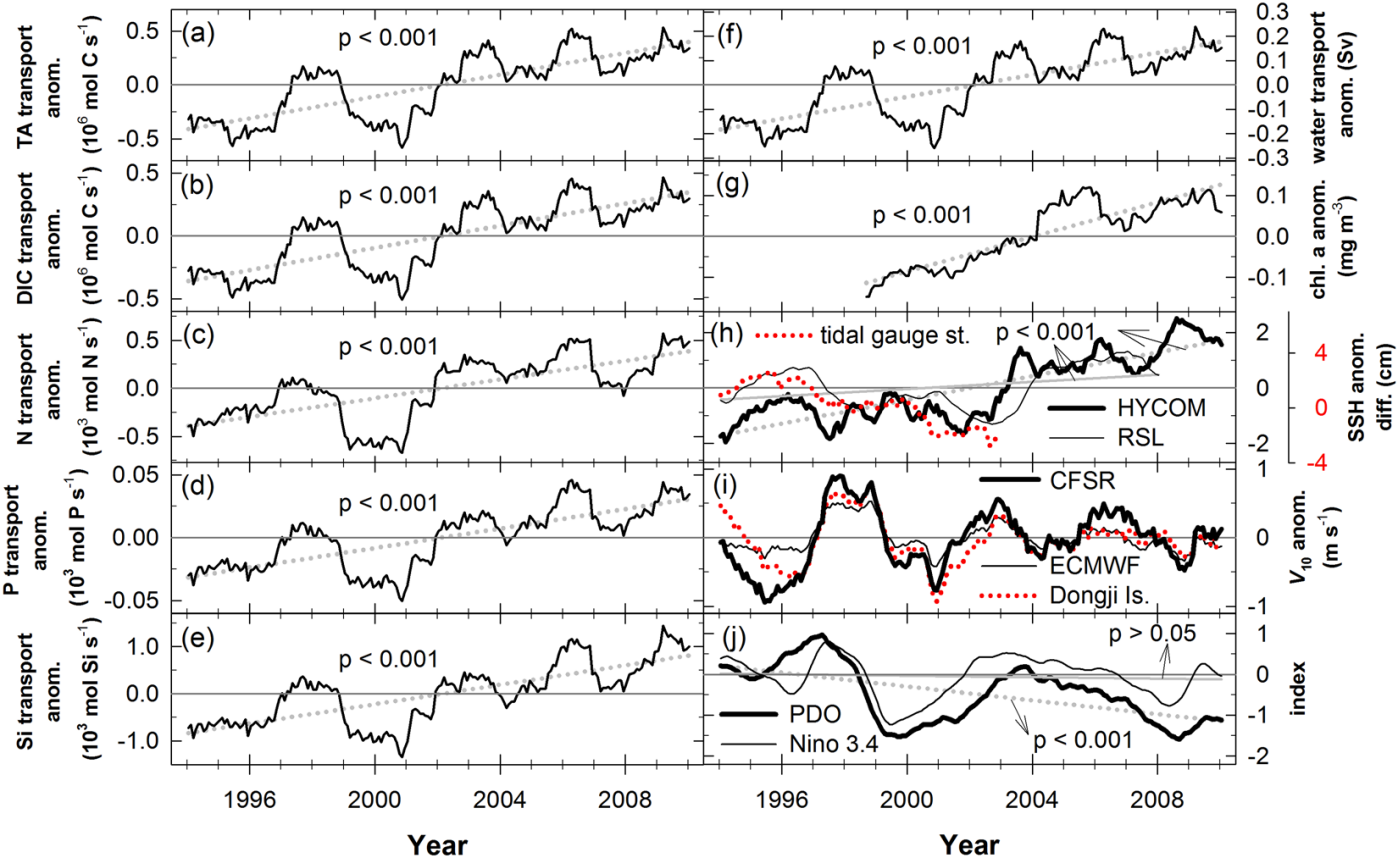
Twenty-four-month moving average time series of anomalies and their regression lines in (**a**) TA transport, (**b**) DIC transport, (**c**) N transport, (**d**) P transport, (**e**) Si transport, (**f**) water transport, (**g**) satellite chl. a concentration, (**h**) individual SSH difference of the HYCOM, RSL, and the gauge stations (Shanwei - Xiamen) between of the southern and northern entrances, (**i**) wind speed of the CFSR (Climate Forecast System Reanalysis), ECMWF (European Center for Medium-range Weather Forecasts), and the Dongji Island weather station in *v* direction (south-north) in 10 m height, and (**j**) PDO and Niño 3.4 indexes. Dashed and solid lines in (**h**) are regression line of the HYCOM and the RSL (Reconstructed Sea Level), accordingly. Dashed and solid lines in (**j**) are regression line of PDO and Niño 3.4, respectively.

The TS exhibits both annual and interannual variations in the water and chemical transports. To reduce the seasonal pattern, 24-month moving averages of water and anomalous chemical transports were used to identify their long-term patterns (Fig. 4a–f). The shapes of anomalous water and chemical transports are similar. Overall, the water and chemical transports have increased significantly from 1993 to 2010. Other long-term research has identified a strengthened TSC, increasing from 1999 to 2009^50^.

To determine the influence of increasing nutrient transports in the TS, satellite chlorophyll *a* data around the boundary between the TS and the ECS are used to the evaluate long-term biological activity there (Fig. 4g). The 24-month moving average anomalous of satellite chlorophyll *a* data were used to reduce any system error and seasonal influences. The increase in the long-term chlorophyll *a* anomalies may reflect enhanced anomalous nutrient transports. The factors that control the water transport in the TS are local wind speed and the northward pressure gradient, and the latter was suggested as the major factor for the northward transport^35,51–53^. The northward pressure gradient arises from the difference of the sea surfaces height (SSH) between the southern and northern parts in the TS. Data of monthly SSH difference and southerly wind speed were used to capture 24-month moving average anomalies (Fig. 4h,i). The anomalous SSH difference between the southern and northern entrances in the TS increases over time, and is in line with the anomalous water transport in the TS. The long-term trend of the southerly wind speed didn’t change with time. However, the influences of wind were still significant for water transport especially during 1995–2001 (Fig. 4h,i). To discuss the relations between the mentioned factors, the regression formula of transport was derived by using the monthly water transport, local southerly wind speed, and SSH difference between the southern and northern entrances in the TS from 1993–2010.

$$\begin{array}{c}{\rm{water}}\,{\rm{transport}}={\rm{wind}}\,{{\rm{speed}}}_{({{\rm{m}}{\rm{s}}}^{-1})}\times 0.2006\pm 0.0038+\\ {\rm{SSH}}\,{\rm{diff}}{.}_{({\rm{cm}})}\times 0.0925\pm 0.0058+0.204\pm 0.127\\ {\rm{Adjusted}}\,{{\rm{R}}}^{{\rm{2}}}=0.93\end{array}$$

Ranges of wind speed (Climate Forecast System Reanalysis, CFSR) and the SSH difference during 216 months were from −14.0 to 6.3 m s^−1^ (negative: northerly; positive: southerly) and from 12 to 31 cm, respectively. In other words, the variation ranges of wind-speed-affected and SSH-difference-affected water transport were from −2.8 to 1.3 Sv and from 1.1 to 2.9 Sv, respectively. Overall, the SSH difference contributed most of the northward transport, and that is consistent with the published studies^32,35,51,52^. The constant, 0.204 ± 0.127, in the equation may represent mainly the contribution to the northward pressure gradient from outside of the TS, and that will need further studies to explain. For the long-term variations, the anomalous southerly wind speed didn’t vary clearly and the anomalous SSH_HYCOM_ difference increased by 4 cm from 1993 to 2010. Consequently, the water transport was enhanced by 0.37 Sv mainly due to the increasing anomalous SSH difference. Note that the range of increased SSH difference from the dataset of Reconstructed Sea Level^54^ (RSL, 1993–2008) is from −0.5 to 0.5 cm, and the rising trend is consistent with the result of HYCOM. The time span of the tidal gauge station record (Shanwei, Xiamen) is only from 1993 to 2003, and the data of HYCOM and RSL in this time period didn’t show increasing trends (Fig. 4h). The 24-month moving average of the PDO index shows a decreasing trend (Fig. 4j), and that may be related to the increasing SSH difference^55,56^.

## Conclusions

The TS is an important path for two marginal seas, the ECS and the SCS. The highest northward water and inorganic carbon transports are in summer, but for nutrients are from April to June. The average annual water and chemical transports are 1.42 ± 0.16 Sv, 3.21 ± 0.37 mmol C s^−1^, 2.78 ± 0.32 mmol C s^−1^, 1.04 ± 0.43 kmol N s^−1^, 0.19 ± 0.033 kmol P s^−1^, and 4.22 ± 0.86 kmol Si s^−1^, respectively. The northward nutrient transports through the TS with a relatively low N/P ratio contribute to the biological uptake in the P-limited ECS. On the other hand, the southward excess N from the CCC may support 5–8% of the primary production in the northern SCS in December. From 1993 to 2010, increasing water and chemical transports through the TS have enhanced the chlorophyll *a* concentrations in the southern ECS. The increasing difference in the SSHs between the southern and northern of the TS is the major factor for the raising water transport. The strengthened northward current with increased P transport from the TS to the ECS promotes phytoplankton growth. These variations may also be related to the PDO index. The ultimate result is that the riverine P transport is less than the total supplied P transport from the TS.

## Methods

### Physical data

The estimated inorganic carbon and nutrient transports were calculated using the modeled water transport and simulated chemical concentrations. The daily salinity (S), temperature (T), and flow velocity were obtained using the Hybrid Coordinate Ocean Model (HYCOM). The sea surface height (SSH) in the northern and the southern TS were adopted from HYCOM (N: 119–121°E, 25–25.5°N; S: 116.5–120.1°E, 22.6–23.1°N) and Reconstructed Sea Level (RSL) Version 1 with 0.5° × 0.5° resolution^54^ (N: 119.5–121°E, 25–25.5°N; S: 116.5–120°E, 22.5–23°N; 1993–2008). Monthly averaged wind speed data in the Taiwan Strait were obtained from the Climate Forecast System Reanalysis (CFSR) and European Center for Medium-range Weather Forecasts (ECMWF) with a spatial resolution of 0.25° × 0.25° (119.66°E, 23.26°N) and 0.125° × 0.125° (≈117–121.5°E, 22–26°N), respectively. The wind force in the HYCOM system is the CFSR.

To evaluate the S and T values in the model, *in situ* S and T data were compared with the values from the model at the same depth and similar locations. The *in situ* S and T data were collected in the range 116–121.5°E and 22–26.5°N during 1993–2012, from a total of 17584 casts that were adopted from the Ocean Data Bank (http://www.odb.ntu.edu.tw/). Supplementary Fig. S1 displays the seasonal averaged differences and standard deviations between the HYCOM results and the measured values. Since the deepest depth in the TS is approximately 150 m, only S and T data for depths from between 0 to 150 m depth were considered. The mean differences (HYCOM result – measured value) in S and T are 0.10 ± 0.07 and −0.17 ± 0.30 °C, respectively. The seasonal S-averaged differences in winter, spring, summer, and autumn are 0.06 ± 0.05, 0.10 ± 0.05, 0.15 ± 0.08, and 0.10 ± 0.07, respectively. Generally, the HYCOM salinity values are higher than measured data, and the S differences decrease from 0 m to 150 m in depth. The larger S differences in summer, which is the raining season, than in other seasons may be caused by the underestimated fresh water input. The seasonal T-averaged differences in winter, spring, summer, and autumn are −0.32 ± 0.06, −0.31 ± 0.21, 0.08 ± 0.39, and −0.13 ± 0.22, respectively. Most of the HYCOM temperature values are lower than the CTD data except in the ranges of 15–65 m depth in summer and in the 30–70 m depth range in autumn.

### Chemical data

Two horizontal cross-sections across the TS along 23.04 and 24.96°N were obtained to evaluate the variation in the exchanges among water masses in the northern TS and the southern TS (see Supplementary Fig. S2). A total of 2096 bottle samples were obtained using a CTD/Rosette sampler during 57 cruises (1995–2014) in the range 116–121.5°E and 22–26.5°N (see Supplementary Fig. S2). The concentrations of TA, DIC, N, P, and Si were analyzed using published methods. N concentration was determined using the pink azo dye method, with a precision of approximately ± 1 and ± 3% at 35 and 1 μmol kg^−1^, respectively^57^. P concentration was analyzed using the molybdenum blue method with a precision of approximately ± 0.5 and ± 3% at 2.5 and 0.1 μmol kg^−1^, respectively^58^. Si concentration was obtained using the silicon molybdenum blue method with a precision of around ± 0.6 and ± 2% at 150 and 5 μmol kg^−1^, respectively. DIC concentration was measured using acidified samples, and then quantifying the produced CO_2_ gas with an infrared gas analyzer (AS-C3 Apollo Scitech), a single operator multi-parameter metabolic analyzer (SOMMA), or a coulometric detector. Analytical precision was ± 0.1%, and all measurements were calibrated with certified reference material (A. G. Dickson from Scripps Institution of Oceanography). TA was analyzed using the open-cell method of potentiometric titration at 25 °C with a precision of ± 0.1%^59–61^. Supplementary Table S1 lists the 57 cruises.

### Regression formula

To estimate concentrations of chemicals, second-order polynomial regression equations were derived from *in situ* S, T, and measured chemical concentrations following the method of Huang, *et al*.^30^. Considering different water masses in the TS, empirical formulas for chemical concentrations were acquired using seasonal measured physical and chemical data at various depths. Supplementary Table S2 lists the coefficients of regression fits, adjusted coefficients of determination, and residual standard errors. The subsurface seawater (below 81 m) along 23.04°N was described using a different set of formulas. The range of individually adjusted determination coefficients between the simulated values of chemical concentrations and the measured values is 0.62–0.96, and which represents the extent to which the model captured variabilities in the measured data. Fig. S3 displays the integrated coefficients between measured and simulated chemical parameters of which the latter were calculated using the mentioned equations and *in situ* S and T. Overall, the determination correlations demonstrate that the simulated performances of chemical values expressed more than 75% of measured chemical data. Then, the daily averaged transports from 1993 to 2010 were estimated, and then averaged to provide monthly data.

### Satellite data

Chlorophyll *a* data were adopted from the Ocean Colour Climate Change Initiative (OC-CCI) of the European Space Agency (https://www.oceancolour.org) in the range 120.8–121.4°E, 25.8–26.4°N. Version 3.1 of OC-CCI merged MERIS (MEdium Resolution Imaging Spectrometer), Aqua-MODIS (Aqua-Moderate Resolution Imaging Spectroradiometer), SeaWiFS (Sea-Viewing Wide Field-of-View Sensor) and VIIRS (Visible Infrared Imaging Radiometer Suite) data.

### Data from gauging stations

The monthly sea level height data of the tidal gauge stations (Supplementary Fig. S2) in Kanmen (121.23°E, 28.08°N; 1993–2012), Xiamen (118.07°E, 24.45°N; 1993–2004), Shanwei (115.35°E, 22.75°N; 1993–2003), and Waglan Island (114.30°E, 22.18°N; 1993–2012) were obtained from the Permanent Service for Mean Sea Level (PSMSL, http://www.psmsl.org/) and Ding *et al*.^62^. For constructing the time series of sea level data in an individual station and comparing data among different stations, the PSMSL used a common datum, revised local reference (RLR), which is generally 700 cm below mean sea level. The local wind speed data in Dongji Island (119.66°E, 23.26°N) were obtained from the Central Weather Bureau (https://www.cwb.gov.tw/).

### Validations for model sea surface height and water transport

The locations of SSH of HYCOM and the tidal gauging station were mentioned in Method 1. Physical data and 5. Data from gauging stations. Supplementary Fig. S4a,b show the correlations between SSH_HYCOM_ and the data from tidal gauging stations. The data from Kanmen and Xiamen stations show consistent pattern with the HYCOM data near the N section. For the S section, the data from the Shanwei station have higher coefficient of determination with the HYCOM data than from the Waglan Island station, which may reflect more influence from the SCS than from the TS.

Compared with the measured and modelled HYCOM water transports, HYCOM results are similar to the measured data. However, HYCOM results seem to overestimate the southward transport in January and December 2011 (see Supplementary Fig. S4c). In the Interannual trend section, the south-north wind speed and the SSH difference are two factors in our water transport formula. Meanwhile, we also adopted the data from tidal gauging stations to verify HYCOM results. In the Interannual trend section (Results and Discussion), the two factors in the water transport formula are south-north wind speed and the SSH difference. Therefore, we adopted the data in the weak wind speed season (the Southwest monsoon; wind speed_Dongji Is._ ranging from −4.2 to 2.6 m s^−1^) and in the two lowest averaged-wind speed_Dongji Is._ months (August: 1.5 m s^−1^; May: −2.4 m s^−1^). Supplementary Fig. S4d shows the positive correlation between the HYCOM water transport and the SSH (Shanwei – Xiamen). The highest coefficient of determination between the HYCOM water transport and the measured SSH difference was in the lowest wind speed period, confirming that the HYCOM results are in line with the measured data.

### Contribution of predictor variables

To evaluate the contribution of S and T to empirical formulas for chemical concentrations, correlations between stimulated chemical values and different prediction parameter(s) were compared; a higher value of correlation represents more dominance over the stimulated chemical values. The highest correlation value is the combined variables with interaction factor among signal variable and combined variables. The value of S more strongly affects TA, whereas T more strongly affects DIC. The nutrients in the surface layer are affected more by S, whereas those below 81 m are more affected by T (see Supplementary Table S3). Supplementary Fig. S5 displays the measured chemical concentrations as well as differences in the measured values and the estimated data with different flow speeds. As we mentioned in the “Physical data” section, the HYCOM salinity (temperature) values are higher (lower) than measured data. The overestimated S causes TA to be overestimated and nutrient concentrations to be underestimated. The underestimation of T is responsible for the overestimation of DIC and nutrients concentrations. The overestimated TA, overestimated DIC, and underestimated nutrient concentrations in the upper layer may be more influenced by the S data than by the T data. Supplementary Fig. S5 shows small difference between the averaged measured and estimated data.

It is worth noticing that there are two exceptions: (1) the ranges of 15–65 m in depth in summer and (2) the 30–70 m depth range in autumn. These two exceptions are with overestimated S and overestimated T, and this condition would tend to underestimate the nutrient concentrations than in other situations.

### Assumptions made in the regression formula

The following assumptions are made. (1) The biological production and consumption do not vary within a season for fixed S and T; (2) the riverine TA, DIC, N, P, and Si concentrations are fixed throughout each season for 20 years, and (3) any effect of variations of air temperature is negligible.

It is also worth noticing that the rising domestic waste and fertilizer use also increased by 20% of in N and P concentrations in the Yangtze River estuary in the 2000s than in the 1990s^16,63^, and the increasing level was reduced in the high salinity sea waters. Since most of our chemical data were collected from 2000–2014, the regression formula for N and P may overestimate the southward transport of N and P concentrations from 1993–1999. Assuming that the salinity in the Yangtze River estuary were around 15–20, the overestimated percentage would be reduced to 7–13% of N and P concentrations or even lower in the CCC because of biological consumption in the shelf area.

## Supplementary information

Supplementary Information

## Data Availability

All data generated or analyzed during this study are included in this published article (and its Supplementary Information files).
