# Supplementary material for: East China Sea increasingly gains limiting nutrient P from South China Sea
Source: Sci Rep. 2019 Apr 4;9:5648. doi: 10.1038/s41598-019-42020-4 (PMC6449353; doi:10.1038/s41598-019-42020-4)
Supplement: Supplementary file 1 — Supplementary Information [file 41598_2019_42020_MOESM1_ESM.pdf]

East China Sea increasingly gains limiting nutrient P from South China Sea

Ting-Hsuan Huang, Chen-Tung Arthur Chen, Jay Lee, Chau-Ron Wu, You-Lin Wang, Yan Bai, Xianqiang He, Shu-Lun Wang, Selvaraj Kandasamy, Jiann-Yuh Lou, Ben-Jei Tsuang, Hsien-Wen Chen, Ruo-Shan Tseng, Yiing Jang Yang

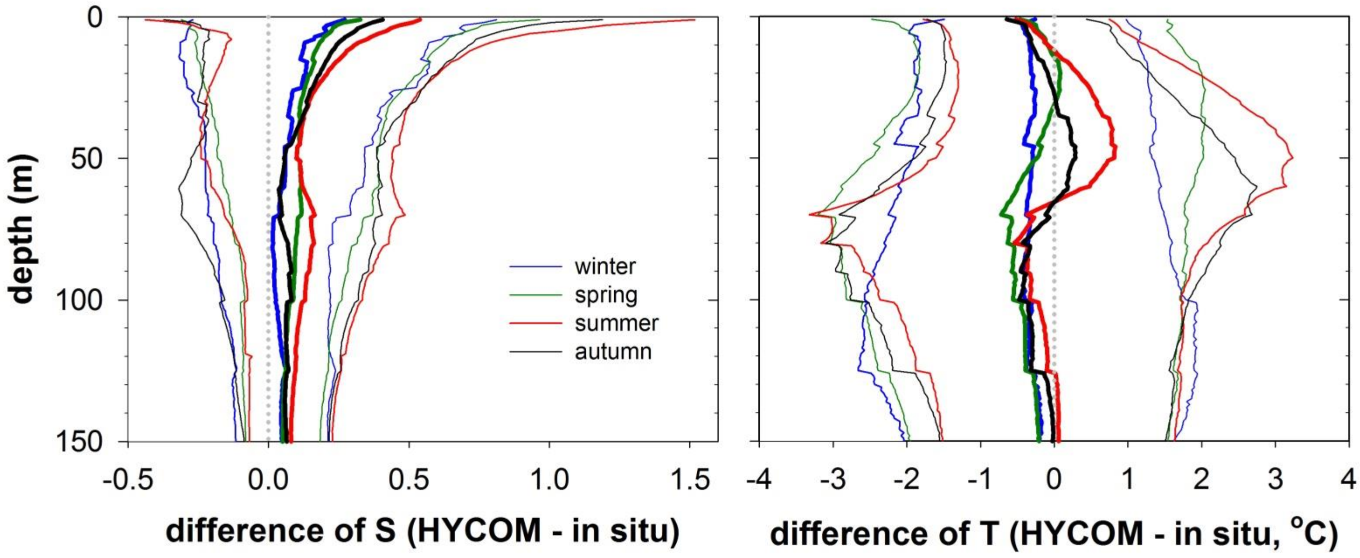

Figure S1 Differences in S (a) and T (b) between HYCOM and measured values at various depths among various seasons. Blue, green, red, and black thick lines represent averaged differences and the area between thin color lines indicates the error range in winter, spring, summer, and autumn, respectively.

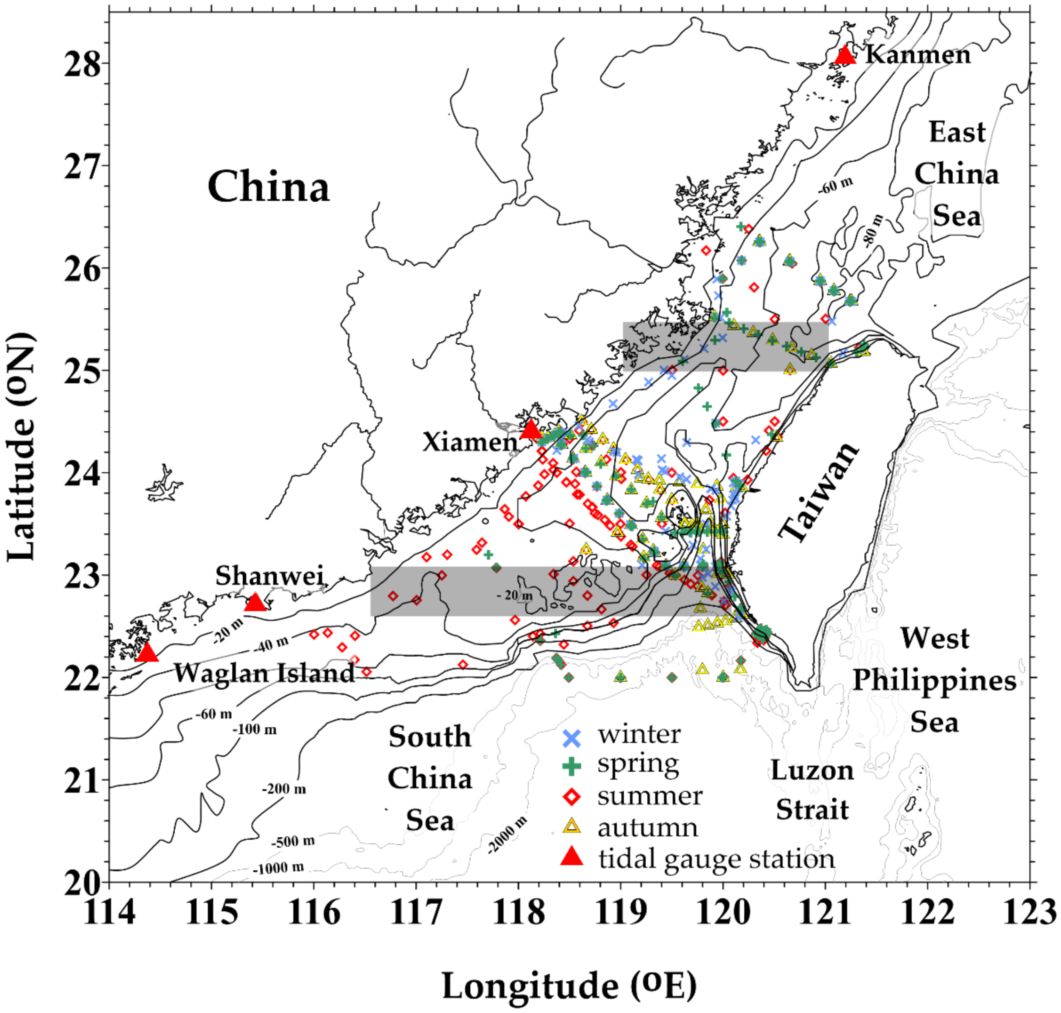

Figure S2 Sampling area. Blue 'x's represent winter cruises; green crosses represent spring cruises; red diamonds represent summer cruises; yellow triangles represent autumn cruises; and red triangles represent tidal gauge stations. The grey rectangles mean the adopted SSH area in the northern and the southern TS.

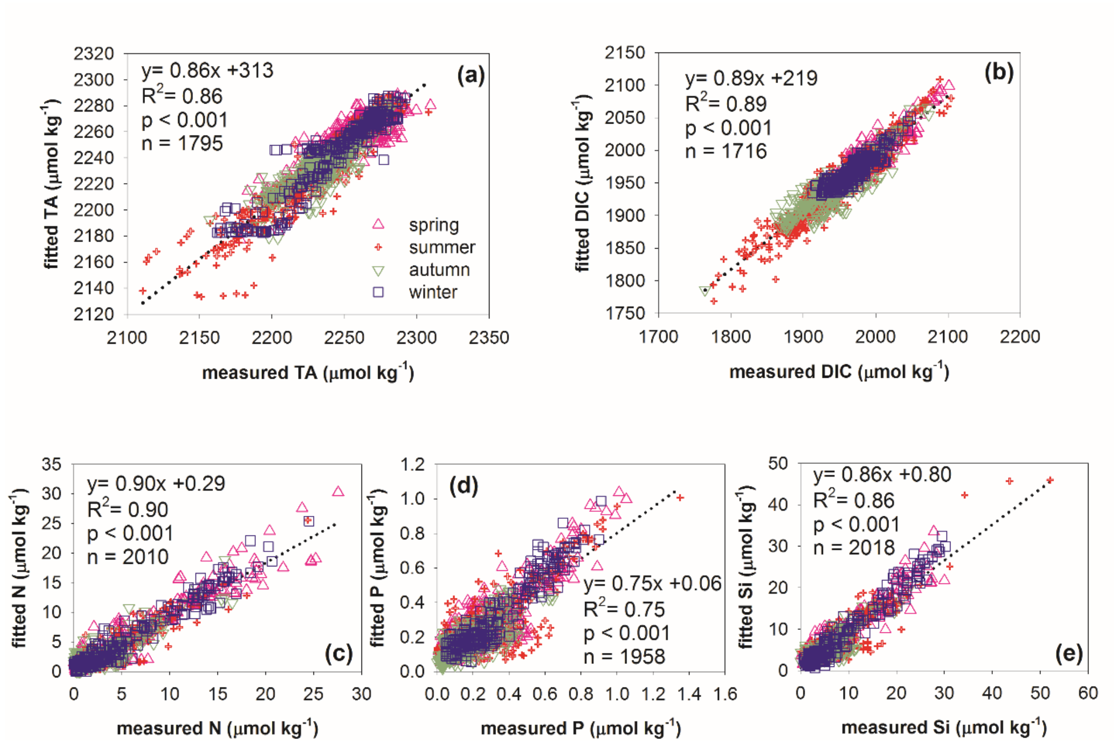

Figure S3 Correlations between fitted and measured concentrations of (a) TA, (b) DIC, (c) N, (d) P, and (e) Si. Salinity and temperature data were adopted from CTD. The symbols of pink triangle, red cross, green inverted triangle, and blue square represent data from spring, summer, autumn, and winter, respectively.

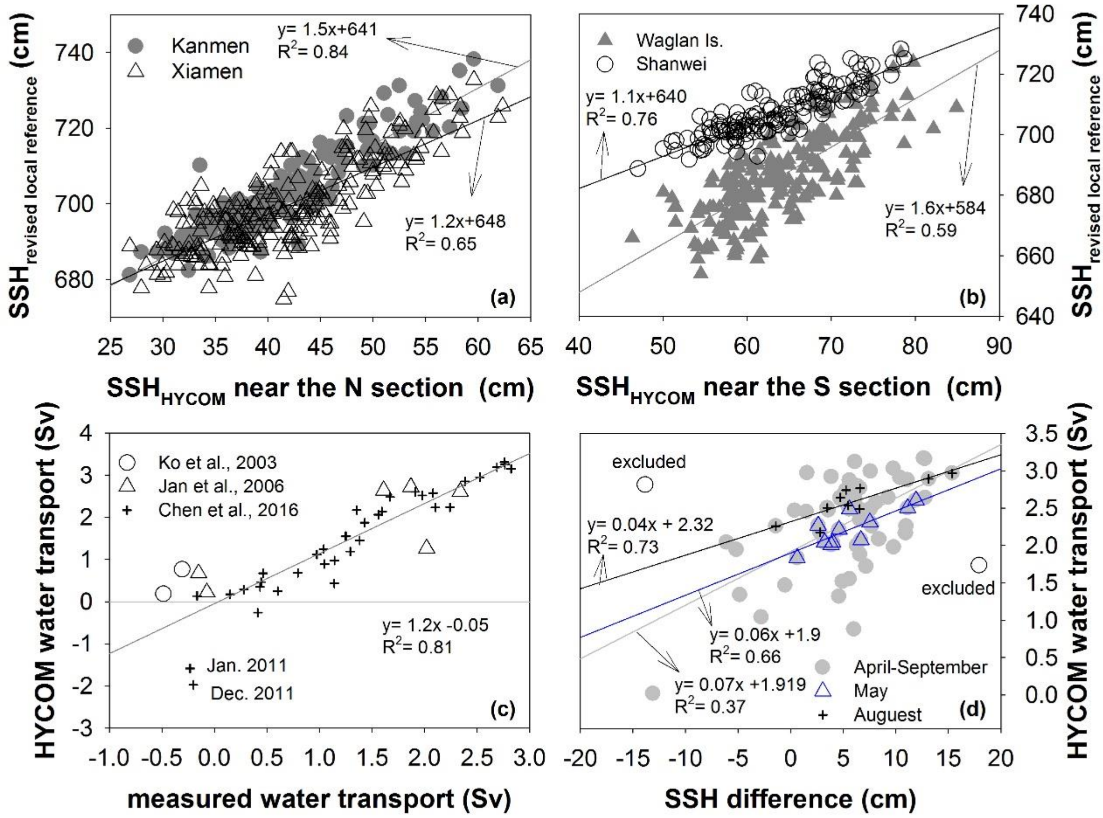

Figure S4 Correlations between $SSH_{revised\ local\ reference}$ and SSH_{HYCOM} near (a) the N section and (b) the S section HYCOM water transport versus (c) measured water transport in published studies and (d) SSH difference between Shanwei and Xiamen stations.

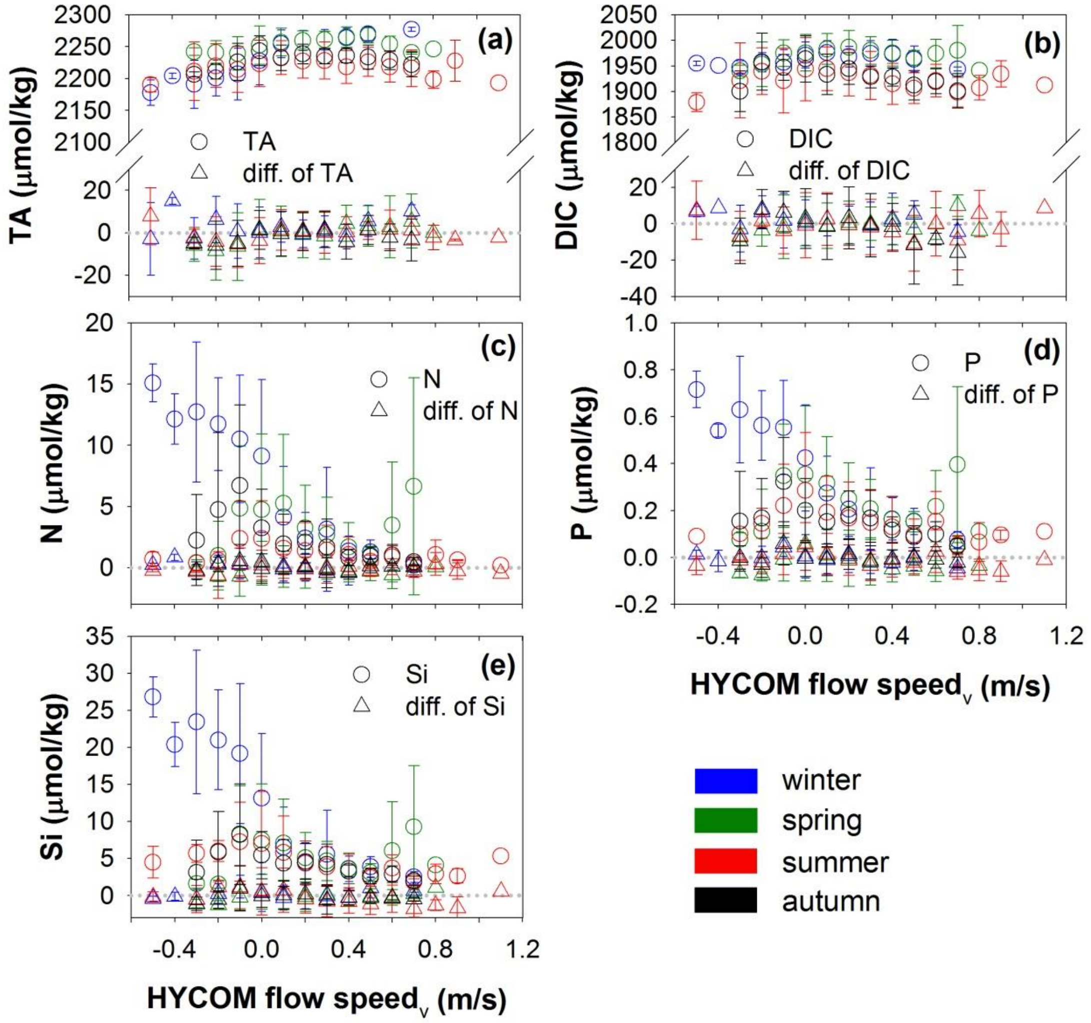

Figure S5 Measured chemical concentrations and the differences between fitted and measured concentrations of (a) TA, (b) DIC, (c) N, (d) P, and (e) Si versus south-north flow speed. Positive and negative values represent the northward and southward fluxes, respectively. Salinity and temperature data used in the formulas were adopted from HYCOM.

Table S1 List of 57 cruises from 1995 to 2014.

Cruise	Date	Cruise	Date
OR1-434	Oct. 26-Nov. 2 1995	OR3-1041	Mar. 1-2 2005
OR3-403	Dec. 20-21 1997	OR2-1268	Mar. 9-10 2005
OR3-551	Aug. 16 1999	OR2-1312	Oct. 6-7 2005
OR3-560	Sept. 11-12 1999	OR3-1105	Oct. 26-27 2005
OR3-632	Jun. 10-11 2000	OR3-1146	Apr. 20-21 2006
OR2-738	Mar. 2-3 2001	OR2-1349	Apr. 29-30 2006
OR3-721	Aug. 6-7 2001	OR3-1161	Jun. 29-30 2006
OR2-806	Aug. 27-28 2001	OR2-1379	Sept. 24-26 2006
OR1-631	Dec. 3-8 2001	OR3-1206	Mar. 5-7 2007
OR3-755	Feb. 25-28 2002	OR2-1419	Mar. 13-14 2007
OR3-776	May. 7-10 2002	OR1-861	Apr. 5-12 2008
OR3-791	June. 24-26 2002	OR1-873	Jul. 30- Aug. 6 2008
OR1-653	Aug. 7-10 2002	OR3-1336	Nov. 25 2008
OR2-1034	Oct. 15-16 2002	OR3-1343	Jan. 5-6 2009
OR3-824	Nov. 4-7 2002	OR1-889	Feb. 11-13 2009
OR1-672	Jan. 13-17 2003	OR3-1347	Mar. 1-7 2009
OR3-851	Mar. 12-14 2003	OR2-1629	Apr. 28-May 1 2009
OR2-1082	Apr. 15-16 2003	OR3-1385	Jul. 20-24 2009
OR2-871	Aug. 1-2 2003	OR3-1436	Mar. 16 2010
OR3-896	Aug. 25-28 2003	OR3-1474	Jul. 11-15 2010
OR1-694	Sept. 15-18 2003	OR1-954	Mar. 9-18 2011
OR2-1139	Sept. 15-18 2003	OR3-1546	Jun. 21-27 2011
OR3-901	Sept. 15-18 2003	OR1-979	Oct. 12-16 2011
OR2-1180	Mar. 16-18 2004	OR3-1659	Nov. 25-27 2012
OR2-1182	Mar. 29-31 2004	OR3-1698	Jul. 2-3 2013
OR3-948	Mar. 29-31 2004	OR2-1953	Jul. 7-8 2013
OR1-713	Mar. 29-Apr. 1 2004	OR3-1735	Dec. 6-8 2013
OR3-983	Jul. 6-9 2004	OR2-2024	Jun. 3-5 2014
OR2-1247	Nov. 10-12 2004		

Table S2 Constants in seasonal simulated chemical equation, adjusted R-squared (adj. R^2), and residual standard error (RSE) of simulated chemical equations.

	**equation**		**$z=z0+a*temp.+b*sal.+c*temp.^2+d*sal.^2+f*temp.*sal.$**							
season	parameter		z0	a	b	c	d	f	Adj. R^2	Root-MSE
winter	TA	<81m	4815.7	0.738	-182.089	0.457	3.367	-0.675	0.90	9.6
		>81m	-13253.3	-504.100	1149.209	2.141	-19.507	12.048	0.85	4.5
	DIC	<81m	5199.5	-81.199	-154.953	-0.984	1.547	3.347	0.70	12.5
		>81m	-7146.7	330.862	293.172	0.367	-0.442	-10.415	0.88	7.3
	N	<81m	490.7	-13.252	-18.220	-0.131	0.068	0.546	0.95	1.2
		>81m	-8591.1	119.593	431.284	-0.427	-5.405	-2.998	0.83	0.8
	P	<81m	22.8	-0.510	-0.937	-0.009	0.005	0.026	0.89	0.1
		>81m	-17.7	4.421	-1.553	-0.004	0.061	-0.126	0.81	0.1
	Si	<81m	1104.6	-0.068	-61.543	-0.098	0.827	0.114	0.95	1.8
		>81m	-4581.0	60.155	234.025	-0.243	-2.970	-1.526	0.86	1.2
spring	TA	<81m	-418.2	-28.346	157.168	-0.075	-2.261	0.811	0.67	12.1
		>81m	-37477.0	-222.406	2413.940	0.222	-36.451	6.106	0.76	5.3
	DIC	<81m	-326.9	-68.614	172.654	-0.056	-2.941	1.823	0.82	12.2
		>81m	-23684.3	275.792	1304.263	-0.561	-16.127	-7.794	0.95	9.6
	N	<81m	370.9	-6.899	-15.160	-0.020	0.127	0.216	0.90	1.5
		>81m	-21957.3	-7.968	1286.761	-0.010	-18.819	0.195	0.95	0.8
	P	<81m	16.4	-0.346	-0.684	-0.002	0.006	0.012	0.62	0.1
		>81m	-653.9	1.767	37.117	-0.003	-0.525	-0.051	0.90	0.1
	Si	<81m	526.8	-5.326	-24.849	-0.032	0.274	0.185	0.87	1.9
		>81m	-59624.0	-50.101	3507.188	0.199	-51.434	1.131	0.93	2.0
summer	TA	<81m	12667.8	-120.760	-563.011	0.346	7.777	2.974	0.87	10.2
		>81m	5213.4	-268.308	-43.409	0.063	-1.164	7.654	0.80	4.4
	DIC	<81m	9592.1	-259.245	-261.689	0.811	1.828	6.029	0.89	15.1
		>81m	-59915.6	-400.736	3840.604	0.521	-58.874	10.622	0.90	10.3
	N	<81m	391.8	-4.186	-19.553	0.093	0.301	-0.032	0.67	1.1
		>81m	-5219.8	-57.035	344.163	0.134	-5.513	1.453	0.87	1.1
	P	<81m	0.9	0.379	-0.316	0.002	0.011	-0.016	0.64	0.1
		>81m	-184.7	-0.842	11.684	0.007	-0.179	0.014	0.87	0.1
	Si	<81m	435.4	0.798	-24.086	0.046	0.384	-0.122	0.62	2.8
		>81m	-15813.4	-70.876	970.648	0.287	-14.703	1.652	0.89	1.7
autumn	TA	<81m	11919.6	-283.530	-381.818	0.395	3.160	7.637	0.67	9.7
		>81m	99595.0	-155.155	-5602.374	-0.150	80.579	4.649	0.86	4.5
	DIC	<81m	12379.5	173.770	-759.536	-0.573	13.238	-4.582	0.62	17.7
		>81m	-10414.0	836.505	235.186	-1.380	3.383	-22.999	0.87	13.4
	N	<81m	1963.6	5.326	-119.154	0.032	1.839	-0.222	0.83	1.2
		>81m	8116.8	-53.451	-432.124	0.095	5.769	1.396	0.84	0.9
	P	<81m	15.3	0.310	-1.037	0.001	0.019	-0.0110	0.62	0.1
		>81m	1766.4	-10.332	-95.700	0.010	1.295	0.286	0.74	0.1
	Si	<81m	1408.7	-0.319	-81.139	0.029	1.206	-0.059	0.62	1.9
		>81m	25600.3	-110.322	-1407.740	0.167	19.397	2.941	0.93	0.8

Table S3 R-squared values for equations that consider temperature only, temperature and salinity without interaction, and temperature and salinity with interaction.

season	parameter		sal. only	temp. only	sal. + temp. (with interation)
winter	TA	<81m	0.85	0.25	0.90
		>81m	0.80	0.07	0.85
	DIC	<81m	0.01	0.37	0.70
		>81m	0.39	0.50	0.88
	N	<81m	0.91	0.55	0.95
		>81m	0.02	0.72	0.83
	P	<81m	0.86	0.42	0.89
		>81m	0.03	0.81	0.81
	Si	<81m	0.94	0.41	0.95
		>81m	0.02	0.86	0.86
spring	TA	<81m	0.41	0.20	0.67
		>81m	0.47	0.54	0.76
	DIC	<81m	0.15	0.77	0.82
		>81m	0.38	0.79	0.95
	N	<81m	0.83	0.75	0.90
		>81m	0.05	0.84	0.95
	P	<81m	0.54	0.51	0.62
		>81m	0.11	0.80	0.90
	Si	<81m	0.82	0.68	0.87
		>81m	0.03	0.75	0.93
summer	TA	<81m	0.85	0.46	0.87
		>81m	0.76	0.34	0.80
	DIC	<81m	0.61	0.75	0.89
		>81m	0.42	0.89	0.90
	N	<81m	0.29	0.54	0.67
		>81m	0.28	0.82	0.87
	P	<81m	0.25	0.43	0.64
		>81m	0.24	0.82	0.87
	Si	<81m	0.43	0.14	0.62
		>81m	0.25	0.83	0.89
autumn	TA	<81m	0.48	0.29	0.67
		>81m	0.83	0.53	0.86
	DIC	<81m	0.26	0.58	0.62
		>81m	0.22	0.83	0.87
	N	<81m	0.74	0.28	0.83
		>81m	0.09	0.82	0.84
	P	<81m	0.38	0.41	0.62
		>81m	0.08	0.59	0.74
	Si	<81m	0.47	0.33	0.62
		>81m	0.04	0.86	0.93
